# Pretreatment optimization of the biomass of *Microcystis aeruginosa* for efficient bioethanol production

**DOI:** 10.1186/s13568-016-0320-y

**Published:** 2017-01-07

**Authors:** Muhammad Imran Khan, Moon Geon Lee, Jin Hyuk Shin, Jong Deog Kim

**Affiliations:** 1 Department of Biotechnology, Chonnam Natational University, San96-1, Dun-Duk Dong, Yeosu, Chonnam 550-749 Korea; 2 Research Center on Anti-Obesity and Health Care, Chonnam National University, San96-1, Dun-Duk Dong, Yeosu, Chonnam 550-749 Korea

**Keywords:** Bioethanol, Fermentable sugars, Saccharification, Fermentation, *Microcystis aeruginosa*

## Abstract

Microalgae are considered to be the future promising sources of biofuels and bio products. The algal carbohydrates can be fermented to bioethanol after pretreatment process. Efficient pretreatment of the biomass is one of the major requirements for commercialization of the algal based biofuels. In present study the microalga, *M. aeruginsa* was used for pretreatment optimization and bioethanol production. Treatment of algal biomass with CaO before acid and/or enzymatic hydrolysis enhanced the degradation of algal cells. Monomeric sugars yield was increased more than twice when biomass was pretreated with CaO. Similarly, an increase was noted in the amount of fermentable sugars when biomass was subjected to invertase saccharification after acid or lysozyme pretreatment. Highest yield of fermentable sugars (16 mM/ml) in the centrifuged algal juice was obtained. 4 Different microorganisms’ species were used individually and in combination for converting centrifuged algal juice to bioethanol. Comparatively higher yield of bioethanol (60 mM/ml) was obtained when the fermenter microorganisms were used in combination. The results demonstrated that *M. arginase* biomass can be efficiently pretreated to get higher yield of fermentable sugars for enhanced yield of bioethanol production.

**Electronic supplementary material:**

The online version of this article (doi:10.1186/s13568-016-0320-y) contains supplementary material, which is available to authorized users.

## Introduction

Problems associated with fossil fuels like global warming, environmental pollution and continuous depletion, enforce the scientists to search for renewable, sustainable and ecofriendly alternatives (Hill et al. 2006; Krishna et al. 2001). Biofuels are considered to be the excellent substitute to fossil liquid fuels. Bioethanol is a good alternative for petroleum oils as a transportation fuel while reducing costs, greenhouse gas emissions and waste streams. It can be used alone or can be blended with gasoline as transportation fuels (Rathmann et al. 2010; Schenk et al. 2008; Kaygusuz 2009). Bioethanol can be produced from a number of carbohydrates rich feedstock such as corn, sugarcane, sugar beet and microalgae etc. In the last few decades’ microalgae got considerable interest as feedstock for biofuels production. Microalgae are considered to be the most suitable candidates for biofuels production due to their multisided beneficial aspects such as fast growing rate as compared to terrestrial plants and non-edibility etc. Microalgae have high ability of fixing atmospheric CO_2_ as they efficiently absorbed CO_2_ from industrial exhaust gases, atmosphere and soluble carbonate salts moreover there is no need of arable land for microalgae cultivation (Banerjee et al. 2002; Guschina and Harwood 2006; Singh et al. 2011; Chisti 2007; Hu et al. 2008).

Microalgae produce and accumulate considerable quantities of lipids and carbohydrates which are the valuable raw materials for biodiesel and bioethanol production (Mata et al. 2010). The current cost of biofuels production is not economical and uncompetitive with prices of fossil fuels (Rodolfi et al. 2009). An efficient and cheap feedstock such as biomass of algae may be helpful in reducing the cost associated with biofuels generation (Wijffels and Barbosa 2010) Microalgae as a feedstock attracted much of the attention for biodiesel production (Rodolfi et al. 2009; Miao and Wu 2006). Although the carbohydrates produced by microalgae are also important as raw materials for bioethanol production. However, the carbohydrates produced by microalgae are considered to be insufficient for large scale production of bioethanol. Some microalgae species produce higher carbohydrates quantities as compared to lipids contents, (Deng and Coleman 1999; Dexter and Fu 2009). Also the amounts of carbohydrates can be induced to enhance by nutritional and/or environmental factors to produce a viable algal strain for bioethanol generation (Ho et al. 2013; Chen et al. 2013).

Efficient pretreatment has a great contribution in optimizing the cost associated with bioethanol production. The cost of production is mainly dependent on the pretreatment process (Alvira et al. 2010). Bioethanol production through fermentation is a simple process and need less energy compared to biodiesel production system. The production of bioethanol from microalgae is still under investigation and this technology has not yet been commercialized (Harun et al. 2010a, b). One of the main challenges in the field of biofuels generation is the pretreatment of biomass. In case of bioethanol production from algae, the pretreatment of biomass is required for lysing the cells and liberating the structural and stored carbohydrates to the external media. Most of the carbohydrates are present in polysaccharide form in the algal cell either as structural components or storage materials and need to be transformed into monomeric sugars such as glucose, fructose, galactose etc. These monomeric sugars can be directly converted to bioethanol via fermentation. Successful lysing of algae cells, release and conversion of carbohydrates is the most crucial step for biofuels production. An efficient pretreatment will produce greater amount of fermentable sugars and will results in a good yield of fermentative ethanol (Mosier et al. 2005). The pretreatment of biomass can be processed by different methods such as mechanical methods and acid or enzymatic hydrolysis etc. Some methods are not productive at large scale and commercial levels for example mechanical methods are not suitable for large scale production because of higher energy cost (Hendriks and Zeeman 2009). Manly for bioethanol production the pretreatment of biomass is carried out through acid hydrolysis (with dilute and concentrated acids) and enzymatic hydrolysis to produce fermentable sugars constituents required for fermentation (Saha et al. 2005).

The microalga (cyanobacteria) *Microcystis aeruginosa* is blooming in fresh water in many regions of the world. It synthesizes different types of carbohydrates by photosynthesis and stores in its biomass. The total carbohydrates are about 57% per dry weight of the cell (Kim et al. 2012). In present study *M. aeruginosa* (KMMCC-1135) biomass was used as feedstock for optimization of the pretreatment process and bioethanol production. Efficient pretreatment of the biomass means to released and convert all the stored carbohydrates to fermentable sugars which will aid in the availability of higher quantities of sugars for the producer microorganisms. Our previous studies (Khan et al. 2016) shown that growth rate and carbohydrates synthesis of *M. aeruginosa* can be enhanced by optimizing different culture parameters. Beside extraction of sugars for bioethanol synthesis, the biomass of *M. aeruginosa* can also be used for the extraction of cytotoxic and antimicrobial agents (Ishida et al. 1997). This study will be helpful as a model for optimizing the pretreatment process and bioethanol production with other useful and carbohydrates rich microalgae strains.

## Materials and methods

First *M. aeruginosa* (KMMCC-1135) was cultured in MF media under red LED light of 5000 lx with a continuous supply of 2% CO_2_ (Khan et al. 2016). Conical flasks (500 ml) were used for the culture of *M. aeruginosa*. The flasks were kept in a shaking incubator at 25 °C with 100 rpm. Biomass accumulation was analyzed by measuring OD_660_ of the culture after every 24 h. OD (Optical density) of the culture is directly related to the cell concentrations/biomass (Ho et al. 2013). After two weeks, culture (OD_660_ = 1.4) was directly used as a biomass for pretreatment experiments. 200 ml algae biomass was used in each pretreatment experiment. Whenever lysozyme was added pH of the media was brought to 6.2. Similarly, for invertase action pH was kept 4.5. After acid hydrolysis in each experiment the resulting algae juice was neutralized with 10 M NaOH. After neutralization the reduced sugar quantity in the samples were measured by the method of Miller (1959) with little modifications. 1 ml DNS solution was mixed with 1 ml of the neutralized algae juice in Eppendorf tubes and the mixture was heated at 100 °C on hot block for 10 min. After cooling in cold water, absorbance was checked at 575 nm by spectrophotometer.

## Pretreatment of *M. aeruginosa* biomass

### Acid hydrolysis

*Microcystis aeruginosa* biomass was treated with different molarities of H_2_SO_4_ (1M, 3M, 5M, 7M and 10M). 20 ml of H_2_SO_4_ of each molarity was added to 200 ml of the algal biomass in 500 ml conical flasks. All of the flasks were heated at 150 °C for 30 min. After cooling the hydrolysate was neutralized with 10M NaOH. After neutralization the algae juice was centrifuged and carbohydrates were measured in the sample by the same above method.

### Pretreatment with Tio_2_ and acid hydrolysis

Effect of TIO_2_ on the biomass degradation and assisting the acid hydrolysis process was investigated. In these experiments the algal biomass (200 ml) was first treated with different amounts of TiO_2_ and then subjected to acid saccharification. After addition of TiO_2_, biomass was kept in shaking incubator for mixing for 4 h. After that the biomass was treated with 5M H_2_SO_4_ at 150 °C for 30 min. The hydrolysate was neutralized with 10M NaOH and sugars contents in the centrifuged algal juice was measured through DNS method.

### Lysis with lysozyme

Lysozyme MDL number MFCD00131557 was purchased from sigma Aldrich Korea. Aqueous stock solution was prepared as 0.002 g/10 ml according to the manufacturer instructions. Algae biomass was treated with different volumes of the lysozyme solution (0.2, 0.4, 0.6, 0.8, 1.0, 1.5, 2.0 and 4.0 ml). Working pH for lysozyme reaction was set 6.2. After addition of lysozyme biomass was kept at 50 °C for 8 h in a shaking incubator. After lysozyme treatment the hydrolysate was centrifuged and carbohydrates were measured through DNS reduction method.

### Lysozyme and acid hydrolysis

The combine effect of Lysozyme and acid hydrolysis was investigated. Algae biomass was treated in different patterns with lysozyme and acid. In first attempt the biomass was first treated with lysozyme and then with 5M H_2_SO_4_. In second attempt the biomass was first treated with 5M H_2_SO_4_ and then followed by degradation with lysozyme. Effect of temperature on acid hydrolysis and lysozyme action was also investigated. Acid hydrolysis and lysozyme degradation was done both at high and low temperature. Highest temperature was 150 °C and lowest was room temperature (25 °C).

### Pretreatment with lysozyme, acid hydrolysis and invertase

In this experiments effect of invertase enzyme was tested on the yield of fermentable sugars in the algal juice resulted from lysozyme and acid pretreatment. After acid hydrolysis some carbohydrates remained in polymeric and fermentable form. Sucrases are enzymes which cleave the sugars macromolecules and convert to monomeric fermentable form usually sucrose to glucose (invertase). Invertase, Enzyme Commission (EC) Number 3.2.1.26 was purchased from sigma Aldrich Korea. 0.006 g invertase was directly added to the algal biomass pretreated with acid and lysozyme under the above same method. Biomass after invertase addition was kept in shacking incubator at pH 4.5 and temperature 45 °C for 8 h. Finally, the algae juice was centrifuged and carbohydrates were measured by the same above method.

### Pretreatment with alkaline hydrogen peroxide solution

The effect of alkaline hydrogen peroxide solution on biomass was studied. Aqueous solution of H_2_O_2_ (pH 11.5) was prepared and 20 ml was added to algal biomass for pretreatment. After the addition of H_2_O_2_ the biomass was then kept in a shaking incubator for 4 h. After pretreatment with alkaline H_2_O_2_ solution algae biomass was subjected to acid and enzymatic scarification under different conditions.

### Pretreatment with CaO

Algae biomass was pretreated first with different quantities of CaO. After addition of CaO, biomass was kept in a shaking incubator at 50 °C for 4 h. After treatment with CaO, biomass was treated with different arrangements of lysozyme and acid hydrolysis. In the last biomass was treated with invertase. Acid hydrolysis was performed with 20 ml of 5 M H_2_SO_4_.

### Fermentation

Fermentation was carried out in 500 ml volume conical flask with 200 ml centrifuged algae juice as substrate. For determining the fermentation process with maximum yield of bioethanol, four different types of microorganisms i.e. *Saccharomyces cervevisiea*, *Brettanomyces custersainus*, *Pichia stipites* and *Klebsiella oxytoca* were used for fermenting the algae juice to bioethanol. All this microorganism was obtained from KCTC Korea. First the producer microorganisms were grown in culture media supplemented with yeast extract, malt extract and peptone. For *B. custersainus* CaCO_3_ was also added to the culture media. In order to find out the optimum fermentation with highest yield, the microorganisms were used individually and in combination with equal amount in the fermentation process. Purpose of the combination of the fermenter microorganism were to convert all types of fermentable sugars to bioethanol as these microorganisms are substrate specific and individually they convert only specific type of fermentable sugars to bioethanol. 20 ml culture of each microorganism (OD_660_ = 1.0) in its exponential phase was added to 200 ml centrifuged algae juice for individual fermentation and 5 ml inoculum of each fermenter microorganism were added to the flask of combine fermentation along with their respective culture media. All the fermentation flasks were kept in a shaking incubator at 25 °C at 150 rpm. Samples were taken after every 3 h and were analyzed for alcohol contents according to the method of Seo et al. (2009).

### Statistical analysis

All experiments were performed in triplicates. Data are the Mean values ± standard deviation. ANOVA were used for Statistical analysis. Statistical significance was set at α = 0.05.

## Results

### Acid hydrolysis

**Fig. 1:**
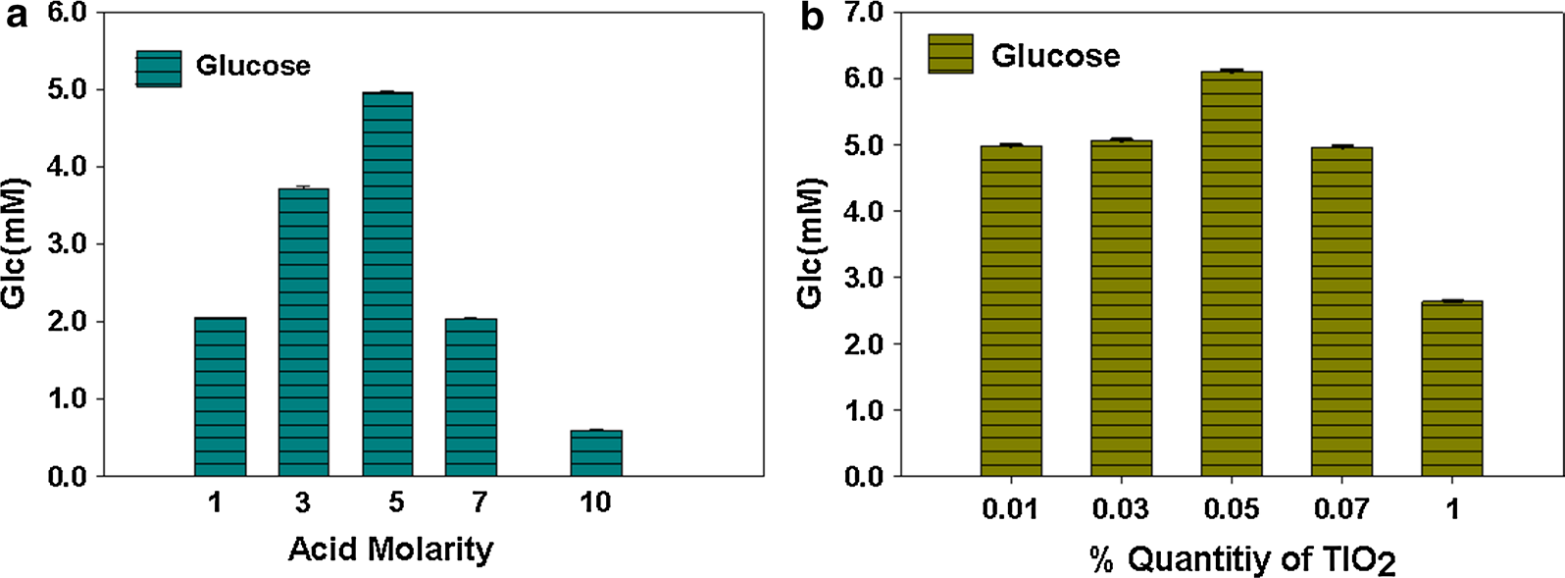
**a** Reduced carbohydrates contents (mM/ml) in the biomass after pretreatment with different strength of H_2_SO_4_ (20 ml). Data represents mean values of different experiments performed in triplicate ± standard deviation. **b** Reduced sugar contents (mM/ml) in the samples of the algae biomass pretreated with different amounts of TiO_2_ followed by acid hydrolysis with 5M H_2_SO_4_. Data represents mean values of different experiments performed in triplicate ± standard deviation

Pretreatment of the algal biomass with acid of different strength resulted in different amounts of the reduced sugars. The highest amount of fermentable sugars was found after biomass hydrolysis with 5 molar H_2_SO_4_ at 150 °C (Fig. 1a). In all other cases comparatively lower sugar contents were obtained. Strength and quantity of acid for a specified amount of biomass directly affect the amount of fermentable sugars (Ho et al. 2013). This indicates that acid molarity (strength) has a great effect on the pretreatment of biomass. The acid strength below and above 5M showed less effectiveness in the pretreatment of algal biomass(200 ml). The lowest amount of carbohydrates was resulted from the biomass treated with 10M H_2_SO_4_. Stronger acid converts the monomeric sugars to furfural, causing reduction in the final yield of fermentable sugars. This indicates that for maximum lysis of algae and production of sugars through acid hydrolysis the amount of acid should be optimized.

### Pretreatment with Tio_2_ and acid hydrolysis

Different quantities of TiO_2_ were used for degrading the biomass and making the target essay for acid hydrolysis. The biomass degradation was found to increase with the increasing quantities of TiO_2_ however the degradative effects was found to decrease when the quantity was increased beyond 0.05%. The highest quantity of sugar content was resulted from the biomass pretreated with 0.05% TiO_2_ followed by acid hydrolysis with 20 ml 5M H_2_SO_4_ (Fig. 1b). This indicated that TiO_2_ is effective in an adequate amount for lysing algal cells.

### Lysis with lysozyme

**Fig. 2:**
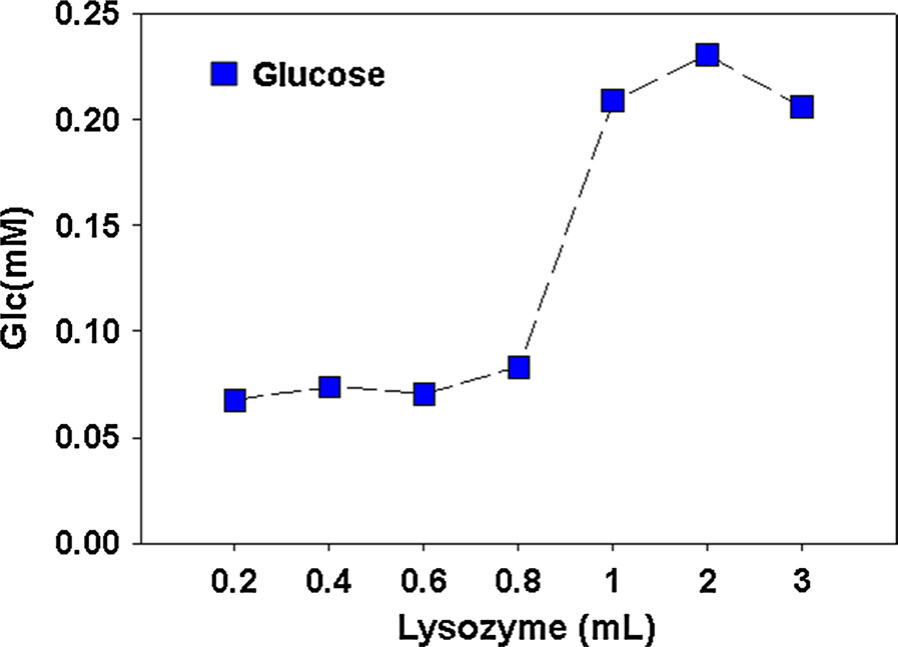
Reduced carbohydrates contents (mM/ml) in the samples resulted from the pretreatment of algal biomass with different quantities of lysozyme. Data represents mean values of different experiments performed in triplicate ± standard deviation

Lysozymes are lysing enzymes having the ability of degrading cell wall of microorganisms like bacteria and microalgae. The mechanisms of lysing involved breaking of linkages between *N*-acetylmuramic acid and *N*-acetyl d-glucosamine residues in peptidoglycan and between *N*-acetyl d-Glucosamine residues in chitodextrin. Hence these enzymes can break the polysaccharides in some extents. Here we tried different amounts of lysozyme solution for degradation of algal cells and releasing of the internal stored sugar contents. The maximum lysis of algae cells was found with 2 ml of lysozyme solution and showed the highest value of reduced sugars than the other volumes of lysozyme (Fig. 2). Hence 2 ml of the lysozyme solution was found to be optimum for the lysis of 200 ml algae biomass.

### Lysozyme and acid hydrolysis

**Fig. 3:**
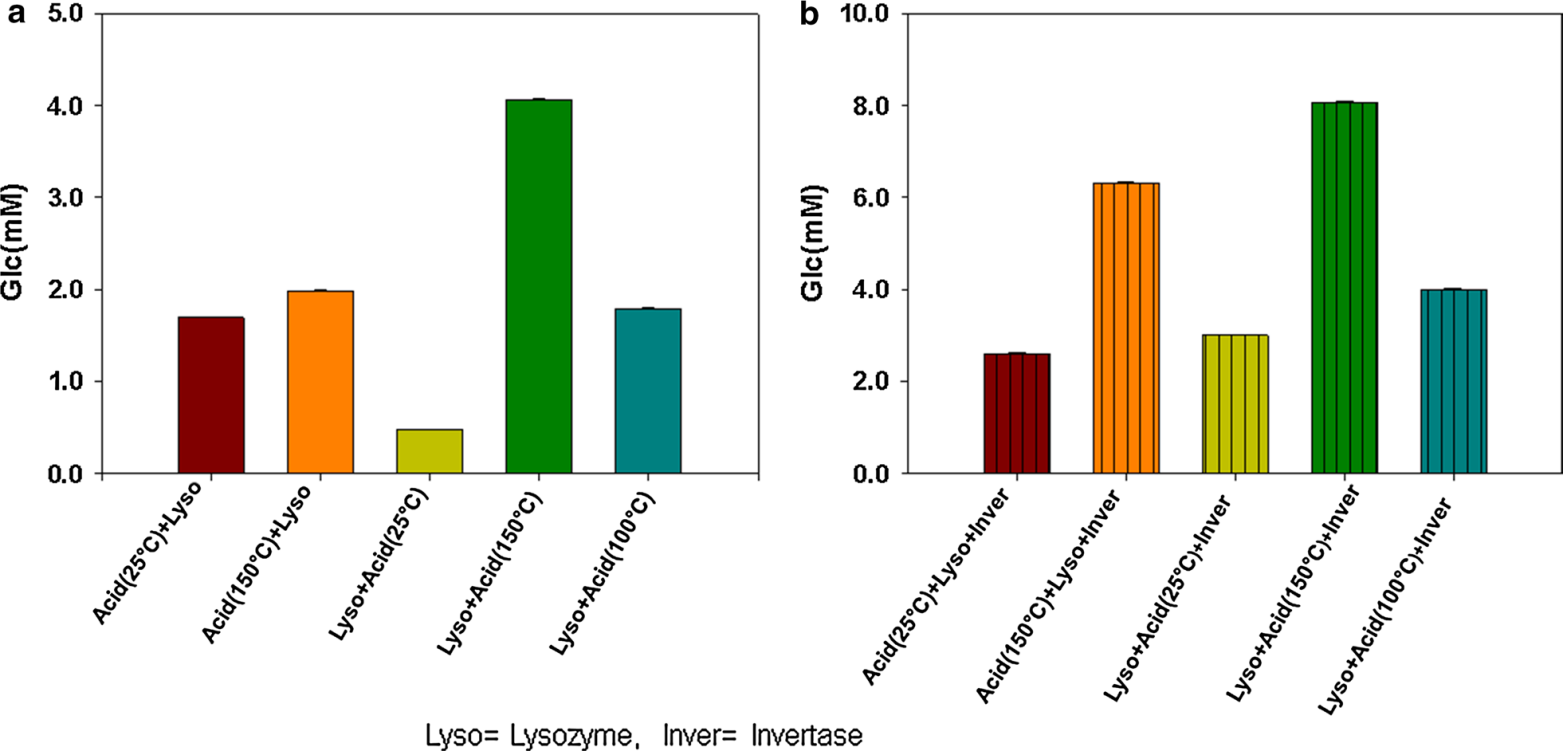
**a** Pretreatment of the biomass with lysozyme (2 ml) and 5M H_2_SO_4_ (20 ml) at different temperatures. In the first two attempts biomass was first treated with acid and followed by hydrolysis with lysozyme at room temperature and 150 °C respectively. In the last three attempts biomass was first treated with lysozyme and then followed by acid hydrolysis at 25, 150 and 100 °C respectively. Data represents mean values of different experiments performed in triplicate ± standard deviation. **b** Reduced sugars contents (mM/ml) in the samples resulted from the pretreatment of algal biomass with lysozyme, acid hydrolysis and saccharification with invertase. Data are expressed as mean of values from experiments in triplicates ± standard deviation

The combination of the lysozyme and acid treatment was made for increasing the sugars yield. In these experiments the highest sugar quantity was obtained as a result of the pretreatment of the biomass with lysozyme followed by acid hydrolysis at 150 °C (Fig. 3a). The pretreatment of the algae biomass first with lysozyme and then with acid did not result in a good yield. Also the higher temperature assisted the pretreatment process with acid and increase the sugars yield.

### Pretreatment with lysozyme, acid hydrolysis and invertase

In order to get higher quantities of sugars from the biomass, various combinations of the lysozyme, acid and invertase were applied to the biomass (Additional file 1: Table S1). The best combination of these reagents which showed the higher degradation and hydrolysis was the pretreatment of the algae biomass with lysozyme followed by acid hydrolysis at 150 °C and finally treatment with invertase. This pretreatment showed the highest yield of sugars among this set of pretreatment experiments (Fig. 3b). Invertases are enzymes which breaks oligosaccharides and disaccharides especially sucrose to monomeric fermentable units. The results indicate that after acidic and enzymatic hydrolysis of the algal biomass treatment with invertase is productive for converting the remaining non fermentable sugars to fermentable units.

### Pretreatment with alkaline hydrogen peroxide solution+ enzymatic hydrolysis + acid hydrolysis

**Fig. 4:**
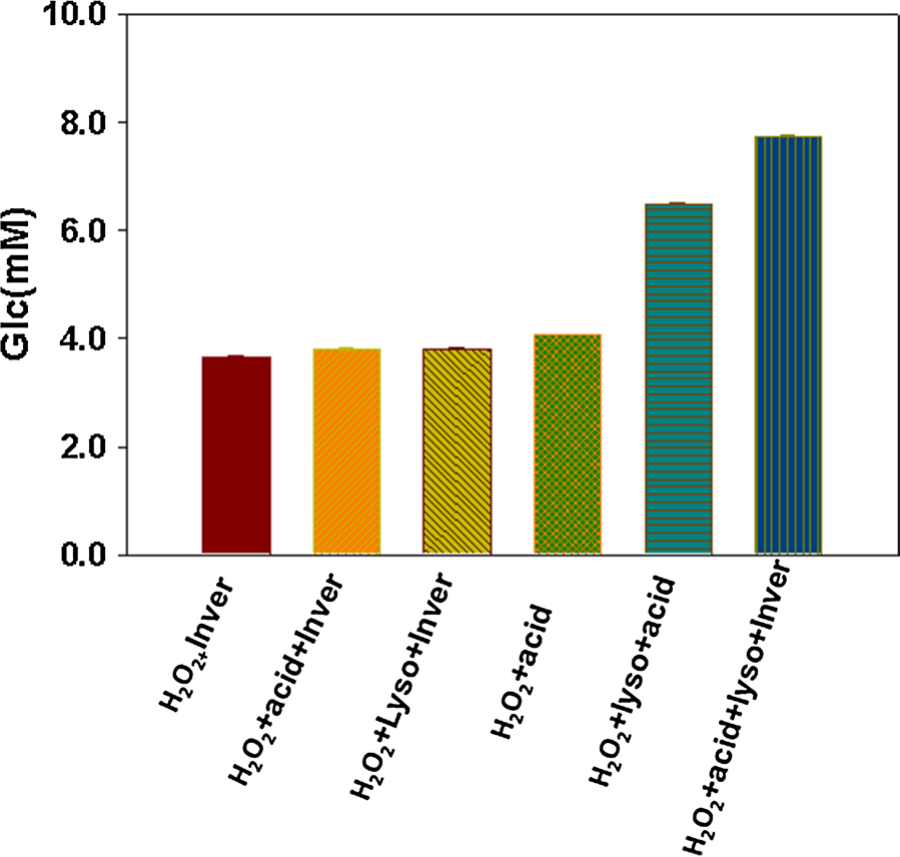
Reduced sugars quantities (mM/ml) in the samples of pretreated biomass of algae with different volume of alkaline H_2_O_2_ solution followed by enzymatic hydrolysis and then acid hydrolysis. Values are the mean of different experiments performed in duplicates ± standard deviation

Effect of the alkaline hydrogen peroxide solution on the degradation of biomass in different combination with lysozyme, acid and invertase were studied (Additional file 1: Table S2). The results obtained showed that H_2_O_2_ is effective in biomass degradation and facilitate the further hydrolysis with acid. Among these group of pretreatment experiments the highest sugar contents was found in the samples resulted from the treatment of biomass first with alkaline hydrogen peroxide solution followed by lysozyme and then with acid hydrolysis and finally with invertase (Fig. 4).

### CaO+ enzymatic hydrolysis+ acid hydrolysis

**Fig. 5:**
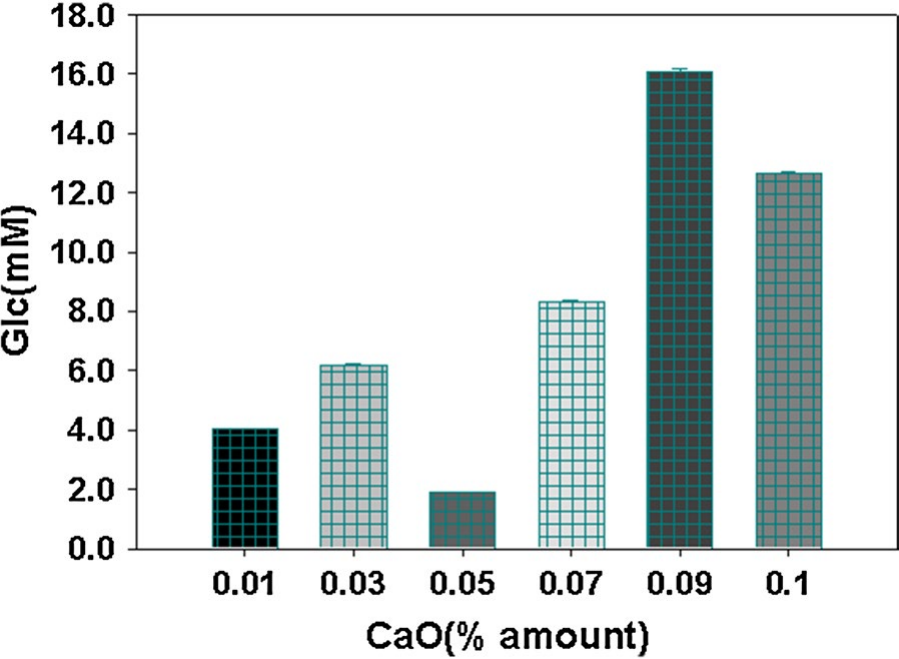
Reduced sugars quantities (mM/ml) in the samples resulted from the pretreatment of biomass with different amount of CaO, With or without lysozyme (2 ml) and acid (5M H_2_SO_4_) and finally with invertase (0.006 g). Data are expressed as mean of values from experiments in triplicates ± standard deviation

The arrangement of lysis experiment for the pretreatment of algae biomass in this group is shown in Additional file 1: Table S3. In this experiments the ability of CaO to lyse the algae cells was investigated and the efficient and appropriate amount of CaO for the biomass treatment was optimized. This method of pretreatment of the algal biomass was found to be most efficient than all other methods used and resulted in highest quantities of fermentable sugars. The highest amount of sugar contents was resulted from the pretreatment of the biomass with CaO followed by lysozyme and acid hydrolysis and then treatment with invertase (Fig. 5).

### Fermentation

**Fig. 6:**
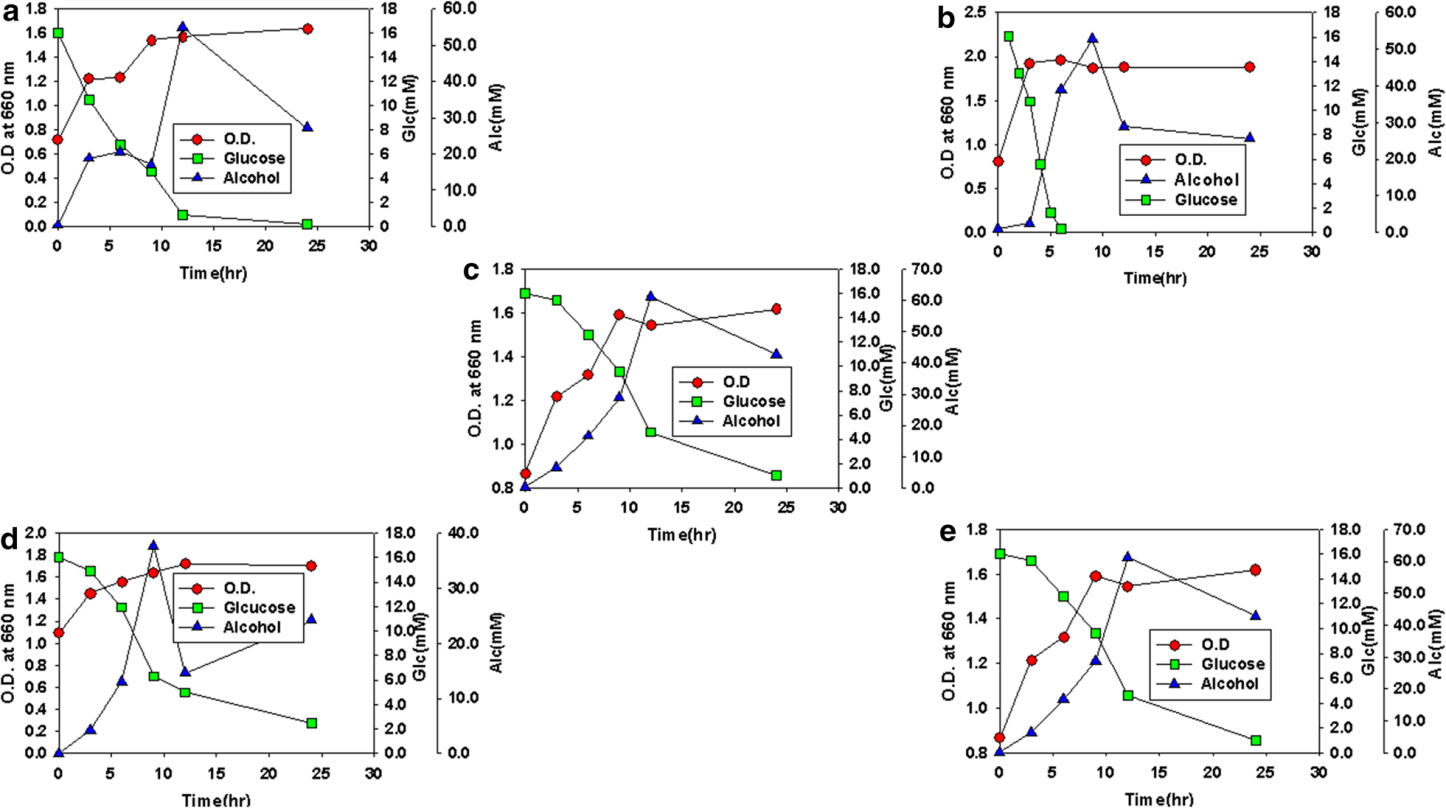
Alcohol contents (mM/ml) in the samples from the fermentation of different microorganism’s species individually and in combination. **a** Fermentation by *S. cervevisiea*. **b** Fermentation by *B. custersainus*. **c** Combine fermentation. **d** Fermentation by *K. oxytoca*. **e** Fermentation by *P. stipites*

The algae juice was then used as substrate for the producer microorganisms to convert to bioethanol. After every 3 h’ samples were taken from the fermentation media and were analyzed for alcohol contents through dichromate oxidation method. Also the reduction in the sugar contents and the increase in the biomass of the fermenting microorganisms were simultaneously measured. The alcohol content was found up and down with respect to time. The highest amount of bioethanol was found in the samples from the combine fermentation of the *S. cervevisiea*, *K. oxytoca*, *B. custersainus* and *P. stipites*. In Individual fermentation higher alcohol content was found in the samples fermented by *S. cervevisiea* (Fig. 6). The highest amount of bioethanol was found 60 mM/ml, higher than the amount of bioethanol in our previous work (Khan et al. 2016).

### Discussion

Biofuels due to its renewable nature had been succeeded in getting the attention of researcher as alternative energy sources to fossil fuels. Using food stuffs as feedstock for biofuels generation are not economical and lead to food and fuel completion. Microalgae are the most suitable candidate for biofuels production at commercial scale. Besides the extraction of lipids and carbohydrates for biofuels production, the algal biomass is also rich source of a verity of antioxidants, antimicrobial and antiviral agents (Pulz and Ross 2004; Pienkos and Darzins 2009). The production of bioethanol requires lysis of algal biomass, liberation and saccharification

of the algal carbohydrates to fermentable sugars that can be readily metabolized and fermented by the fermenting microorganisms. There is no documentation till date about a viable and economic pretreatment method of algal biomass (Rodjaroen et al. 2007).

In present work we used different methods for pretreatment of algal biomass for conversion and liberation of the internal sugars to the external media in fermentable form. In our results we proved that only acid hydrolysis is not sufficient for lysis of algal cells and production of fermentable sugars with good yield. Acid hydrolysis or fruitful in degradation of hemicellulose and separation of pentose from cellulose but it is not productive in case of cellulose degradation. For optimization of acid hydrolysis, we cheeked different strength acid on algae biomass and found that 5M H_2_SO_4_ is most suitable for biomass hydrolysis. Higher quantities are severity of acids leads to degradation of monomeric sugars and conversion to other side products (Miranda et al. 2012) Similarly, acid hydrolysis is productive at high temperature. At low temperature acid cannot breaks bonding between the monomers in polysaccharides. Temperature range of 100–200 °C have been reported for pretreatment of algal biomass depending on reaction time and acid concentration to release monomeric sugars for fermentation (Doan et al. 2012; Harun et al. 2010a, b) Our results indicate that the 150 °C temperature is most suitable for algal biomass degradation with 5M H_2_SO_4_ for 30 min’ reaction time.

Lysozyme was used for destructing the algal wall and fragmenting the cell wall constituents and making easiness for further conversion of the carbohydrates to simpler form by acid or enzyme hydrolysis. The use of lysozyme before acid hydrolysis were found most effective. The amount of monomeric sugars was found highest among all the pretreatment methods when the hydrolysate after lysozyme and acid hydrolysis was treated with invertase. Probably the disaccharides which remained untransformed after the action of lysozyme and acid are further converted by invertase to simplest sugars.

The most productive and efficient pretreatment method among all the tested methods was the treatment of algae biomass first with CaO before enzymatic and acid hydrolysis. CaO effectively lysed the algae cells and makes the further hydrolysis and saccharification process easy.

As a result of the above pretreatment method highest sugar quantiles was obtained.

The algae juice obtained from this pretreatment method was used as sugar substrate for fermentation with the use of microorganisms. Specific Microorganisms have abilities of converting the sugars to ethanol but with different rate. Also the fermenter microorganisms or substrate dependent hence the process of fermentation and yield of bioethanol can be processed efficiently if the substrate is appropriate for the used microorganisms. So we used four different types of microorganisms for fermenting the algal sugars to bioethanol. In our results highest alcohol content was found in the samples from combination fermentation. This may be due the presence of different types of monomeric sugars and the abilities and specificity of the used microorganisms for the substrates. Because these microorganisms are able to specific types of fermentable sugars to ethanol individually e.g. *S. cervevisiea* can fastly and efficiently proceed the fermentation process by converting many types of sugars to ethanol but unable to convert pentosses similarly *B. custersainus* can convert a verities of sugars including pentosses but it rates of conversion and the ability of proceeding the fermentation process is not so efficient as *S. cervevisiea* (Vanderhaegen et al. 2003; Kumar et al. 2009) In the same way *Pichia stipitis* can convert Xylose to ethanol faster than any other microorganisms but cannot converts other fermentable sugars. Hence to proceed the fermentation process efficiently and makes maximum conversion to bioethanol it is the best way to use these microorganisms in combination for fermenting *M. aeruginosa* juice to ethanol as it possesses different types of sugars (Kim et al. 2012).

## Additional file

**Additional file 1: Table S1.** Pretreatment of algal biomass with 20ml acid, followed by 2 ml of lysozyme and Pretreatment of algal biomass with 20ml acid, followed by 2 ml of lysozyme; **Table S2.** Algal biomass pretreatment with different combination of alkaline H_2_O_2_ solution, lysozyme hydrolysis (2ml), acid hydrolysis (5M H_2_SO4) and invertase (0.006 g); **Table S3.** Pretreatment of algae Algal biomass with different combination of CaO, lysozyme hydrolysis (2ml), acid hydrolysis (5M H2SO4) and invertase (0.006 g).

## Abbreviations

OD: optical density
DNS: dinitrosalicylic acid
SD: standard deviation

## References

[CR1] Alvira P, Tomas-Pejo E, Ballesteros M, Negro MJ (2010). Pretreatment technologies for an efficient bioethanol production process based on enzymatic hydrolysis: a review. Bioresour Technol.

[CR2] Banerjee A, Sharma RC, Banerjee UC (2002). *Botryococcus braunii*: a renewable source of hydrocarbons and other chemicals. Crit Rev Biotechnol.

[CR3] Chen C, Zhao X, Yen H, Ho S, Cheng C, Lee D, Bai F, Chang J (2013). Microalgae-based carbohydrates for biofuel production. Biochem Eng J.

[CR4] Chisti Y (2007). Biodiesel from microalgae. Biotechnol Adv.

[CR5] Deng MD, Coleman JR (1999). Ethanol synthesis by genetic engineering in cyanobacteria. Appl Environ Microbiol.

[CR6] Dexter J, Fu PC (2009). Metabolic engineering of cyanobacteria for ethanol production. Energ Environ Sci.

[CR7] Doan QC, Moheimani NR, Mastrangelo AJ, Lewis DM (2012). Microalgal biomass for bioethanol fermentation: Implications for hypersaline systems with an industrial focus. Biomass Bioenerg.

[CR8] Guschina IA, Harwood JL (2006). Lipids and lipid metabolism in eukaryotic algae. Prog Lipid Res.

[CR9] Harun R, Singh M, Forde GM, Danquah MK (2010). Bioprocess engineering of microalgae to produce a variety of consumer products. Renew Sust Energ Rev.

[CR10] Harun R, Danquah MK, Forde GM (2010). Microalgal biomass as a fermentation feedstock for bioethanol production. J Chem Technol Biotechnol.

[CR11] Hendriks A, Zeeman G (2009). Pretreatments to enhance the digestibility of lignocellulosic biomass. Bioresour Technol.

[CR12] Hill J, Nelson E, Tilman D, Polasky S, Tiffany D (2006). Environmental, economic, and energetic costs and benefits of biodiesel and ethanol biofuels. Proc Natl Acad Sci USA.

[CR13] Ho S, Huang S, Chen C, Hasunuma T, Kondo A, Chang J (2013). Bioethanol production using carbohydrate-rich microalgae biomass as feedstock. Bioresour Technol.

[CR14] Hu Q, Sommerfeld M, Jarvis E, Ghirardi M, Posewitz M, Seibert M, Darzins A (2008). Microalgal triacylglycerols as feedstocks for biofuel production: perspectives and advances. Plant J.

[CR15] Ishida K, Matsuda H, Murakami M, Yamaguchi K (1997). Kawaguchipetin B, an antibacterial cyclic undecapeptide from the cyanobacterium *Microcystis aeruginosa*. J Nat Prod.

[CR16] Kaygusuz K (2009). Bioenergy as a clean and sustainable fuel. Energy sources, part A:\recovery, utilization, and environmental effects. Energ Sourc.

[CR17] Khan MI, Lee MG, Seo HJ, Shin JH, Shin TS, Yoon YH, Kim MY, Choi JI, Kim JD (2016). Enhancing the feasibility of *Microcystis aeruginosa* as a feedstock for bioethanol production under the influence of various factors. Biomed Res Int.

[CR18] Kim JD, Chae GW, Seo HJ, Chaudhary N, Yoon YH, Shin TS, Kim MY (2012). Bioalcohol production with microalgae, *Microcystis aeruginosa*. Korean Soc Biotechnol Bioeng J.

[CR19] Krishna H, Janardhan S, Reddy T, Chowdary GV (2001). Simultaneous saccharification and fermentation of lignocellulosic wastes to ethanol using a thermotolerant yeast. Bioresour Technol.

[CR20] Kumar A, Singh LK, Ghosh S (2009). Bioconversion of lignocellulosic fraction of water-hyacinth (*Eichhornia crassipes*) hemicellulose acid hydrolysate to ethanol by *Pichia stipitis*. Bioresour Technol.

[CR21] Mata TM, Martins AA, Caetano NS (2010). Microalgae for biodiesel production and other applications: a review. Renew Sust Energ Rev.

[CR22] Miao XL, Wu QY (2006). Biodiesel production from heterotrophic microalgal oil. Bioresour Technol.

[CR23] Miller GL (1959). Use of dinitro salicylic acid reagent for determination of reducing sugar. Analyt Chem.

[CR24] Miranda JR, Passarinho PC, Gouveia L (2012). Pre-treatment optimization of *Scenedesmus obliquus* microalga for bioethanol production. Bioresour Technol.

[CR25] Mosier N, Wyman CE, Dale BD, Elander RT, Lee YY, Holtzapple M, Ladisch M (2005). Features of promising technologies for pretreatment of lignocellulosic biomass. Bioresour Technol.

[CR26] Pienkos TP, Darzins A (2009). The promise and challenges of microalgal-derived biofuels. Biofuels Bioprod Bioref.

[CR27] Pulz OG, Ross W (2004). Valuable products from biotechnology of microalgae. Appl Microbiol Biotechnol.

[CR28] Rathmann R, Szklo A, Schaeffer R (2010). Land use competition for production of food and liquid biofuels: an analysis of the arguments in the current debate. Renew Energ.

[CR29] Rodjaroen S, Juntawong N, Mahakhant A, Miyamoto K (2007). High biomass production and starch accumulation in native green algal strains and cyanobacterial strains of Thailand. Kasetsart J Nat Sci.

[CR30] Rodolfi L, Chini Zittelli G, Bassi N, Padovani G, Biondi N, Bonini G, Tredici MR (2009). Microalgae for oil: strain selection, induction of lipid synthesis and outdoor mass cultivation in a low cost photobioreactor. Biotechnol Bioeng.

[CR31] Saha BC, Iten LB, Cotta MA, Wu YV (2005). Dilute acid pretreatment, enzymatic saccharification and fermentation of wheat straw to ethanol. Process Biochem.

[CR32] Schenk PM, Thomas-Hall SR, Stephens E, Marx UC, Mussgnug JH, Posten C, Kruse O, Hankamer B (2008). Second generation biofuels: high-efficiency microalgae for biodiesel production. Bioenergy Res.

[CR33] Seo HB, Kim HJ, Lee OK, Ha JH, Lee H, Jung KH (2009). Measurement of ethanol concentration using solvent extraction and dichromate oxidation and its application to bioethanol production process. J Ind Microbiol Biot.

[CR34] Singh A, Nigam PS, Murphy JD (2011). Mechanism and challenges in commercialization of algal biofuels. Bioresour Technol.

[CR36] Vanderhaegen B, Neven H, Coghe S, Verstrepen KJ, Derdelinckx G, Verachtert H (2003). Bioflavoring and beer refermentation. Appl Microbiol Biotechnol.

[CR37] Wijffels RH, Barbosa MJ (2010). An outlook on microalgal biofuels. Science.

