# Supplementary material for: Pretreatment optimization of the biomass of Microcystis aeruginosa for efficient bioethanol production
Source: AMB Express. 2017 Jan 7;7:19. doi: 10.1186/s13568-016-0320-y (PMC5218947; doi:10.1186/s13568-016-0320-y)
Supplement: Supplementary file 1 — Additional file 1: Table S1. Pretreatment of algal biomass with 20ml acid, followed by 2 ml of lysozyme and Pretreatment of algal biomass with 20ml acid, followed by 2 ml of lysozyme; Table S2. Algal biomass pretreatment with different combination of alkaline H2O2 solution, lysozyme hydrolysis (2ml), acid hydrolysis (5M H2SO4) and invertase (0.006 g); Table S3. Pretreatment of algae Algal biomass with different combination of CaO, lysozyme hydrolysis (2ml), acid hydrolysis (5M H2SO4) and invertase (0.006 g). [file 13568_2016_320_MOESM1_ESM.docx]

**Pretreatment optimization of the biomass of** ***Microcystis aeruginosa* for efficient bioethanol production**

**Muhammad Imran KHAN^1^, Moon Geon LEE^1^, Jin Hyuk SHIN^1^, Jong Deog KIM^*1, 2^**

*^1^Department of Biotechnology, Chonnam Natational University, San96-1, Dun-Duk Dong, Yeosu, Chonnam, 550-749, Korea.*

*^2^Research center on Anti-Obesity and Health Care, Chonnam National University, San96-1, Dun-Duk Dong, Yosu, Chonnam, 550-749, Korea.*

*TEL & FAX: +82-61-659-7305,* [pasteur@jnu.ac.kr](mailto:pasteur@jnu.ac.kr)

Muhammad Imran KHAN: imranbiotech1@gmail.com

Moon Geon LEE: caky123@naver.com

Jin Hyuk SHIN: [geobae@biolsystems.com](mailto:geobae@biolsystems.com)

Jong Deog KIM:[pasteur@jnu.ac.kr](mailto:pasteur@jnu.ac.kr)

Corresponding author: Prof. Jong Deog KIM (email: pasteur@jnu.ac.kr)

Table S1. Pretreatment of algal biomass with 20ml acid, followed by 2 ml of lysozyme and 0.006 g of invetase at different temperature

| **1^st^ treatment** | **2^nd^ treatment** | **Temperature** | **Invertase** |
| --- | --- | --- | --- |
| Acid | Lysozyme | Room temperature | 0.006 g |
| Acid | Lysozyme | 150°C | 0.006 g |
| Lysozyme | Acid | Room temperature | 0.006 g |
| Lysozyme | Acid | 150°C | 0.006 g |
| Lysozyme | Acid | 100°C | 0.006 g |

Table S2. Algal biomass pretreatment with different combination of alkaline H_2_O_2_ solution, lysozyme hydrolysis(2ml), acid hydrolysis (5M H_2_SO_4_) and invertase (0.006 g)

| **H_2_O_2_(ml)** | **Lysozyme** | **Acid hydrolysis** | **Invertase** |
| --- | --- | --- | --- |
| 10 | - | - | 0.006 |
| 10 | - | 20ml | 0.006 |
| 10 | 2ml | - | - |
| 10 | - | 20ml | **-** |
| 10 | 2ml | 20ml | 0.006 |
| 10 | 2ml | 20ml | - |

Table S3. Pretreatment of algae Algal biomass with different combination of CaO, lysozyme hydrolysis(2ml), acid hydrolysis (5M H2SO4) and invertase (0.006 g)

| **CaO%** | **Lysozyme** | **Acid hydrolysis** | **Invertase(g)** |
| --- | --- | --- | --- |
| 0.01 | - | - | 0.006 |
| 0.03 | - | 20ml | 0.006 |
| 0.05 | 2ml | - | 0.006 |
| 0.07 | 2ml | 20ml | 0.006 |
| 0.09 | 2ml | 20ml | 0.006 |
| 0.1 | 2ml | 20ml | 0.006 |
